# Supplementary material for: RHΔgra17Δnpt1 Strain of Toxoplasma gondii Elicits Protective Immunity Against Acute, Chronic and Congenital Toxoplasmosis in Mice
Source: Microorganisms. 2020 Mar 1;8(3):352. doi: 10.3390/microorganisms8030352 (PMC7142655; doi:10.3390/microorganisms8030352)
Supplement: Supplementary file 1 [file microorganisms-08-00352-s001.pdf]

SUPPORTING INFORMATION

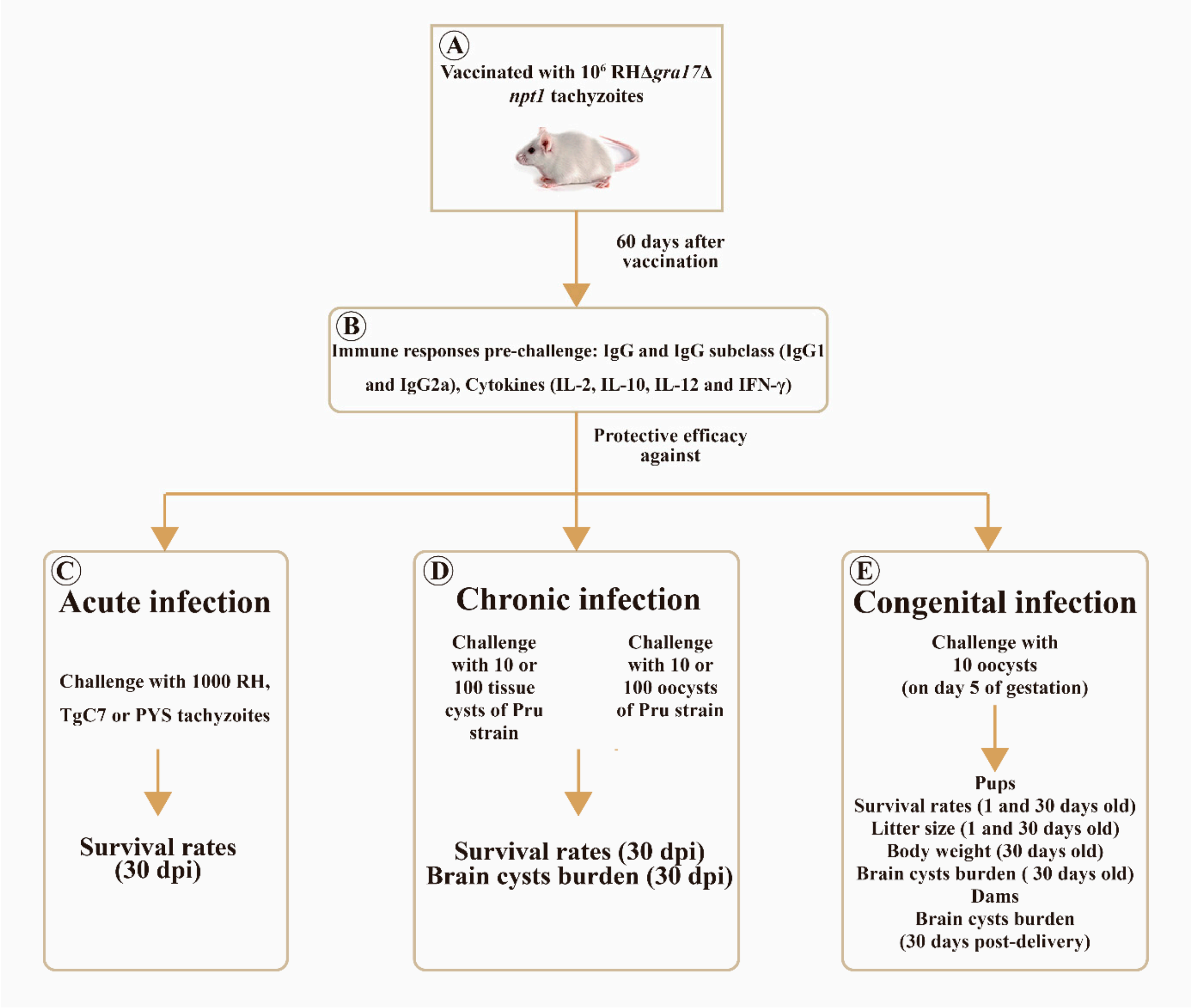

FIGURE S1 | Schematic illustration of the study design. Kunming mice were vaccinated with 10^6 RHΔ*gra17*Δ*npt1* **(A)**. The levels of immune responses **(B)**, and protective efficiency of vaccination against acute **(C)**, chronic **(D)** and congenital **(E)** infection were determined. For simplicity, this schematic shows only the overall design of the study, and more details about the experiments can be found in the "Materials and Methods."

TABLE S1 | General information of sgRNA and primers used in this study.

**Primer**	**Sequence (5' – 3')**
sgRNA-*GRA17*	GACTGTCCCTGAGGACCCAT
KO-*GRA17*-F	CAATCCAGGGACGAACCATT
KO-*GRA17*-R	TCTGCTTCACGGCCATCTT
sgRNA-*NPT1*	GATGAAGGTGCAAGCTCCCG
KO-*NPT1*-F	ACAATGTGGGAAAGTACC
KO-*NPT1*-R	GAAGAGCAGACAAGGAAT
